# Rezafungin Versus Caspofungin in Candidaemia and Invasive Candidiasis: A Post Hoc Pooled Analysis of Phase 2 and Phase 3 Trials

**DOI:** 10.1111/myc.70205

**Published:** 2026-07-16

**Authors:** Alex Soriano, George R. Thompson, Oliver A. Cornely, Matteo Bassetti, Bart Jan Kullberg, Patrick M. Honoré, Hervé Dupont, Jose A. Vazquez, Haihui Huang, Yingyuan Zhang, Sara Dickerson, Laura Cox, Peter G. Pappas

**Affiliations:** ^1^ Department of Infectious Diseases, Hospital Clínic de Barcelona, IDIBAPS University of Barcelona, CIBERINF Barcelona Spain; ^2^ Department of Internal Medicine, Division of Infectious Diseases University of California Davis Medical Center Sacramento California USA; ^3^ The Department of Medical Microbiology and Immunology University of California Davis Davis California USA; ^4^ Institute of Translational Research, Cologne Excellence Cluster on Cellular Stress Responses in Aging‐Associated Diseases (CECAD), Faculty of Medicine University of Cologne Cologne Germany; ^5^ Department I of Internal Medicine, Division of Infectious Diseases, Excellence Center for Medical Mycology (ECMM), Faculty of Medicine University Hospital Cologne Cologne Germany; ^6^ German Centre for Infection Research (DZIF), Partner Site Bonn‐Cologne Cologne Germany; ^7^ University of Cologne, Faculty of Medicine and University Hospital Cologne, Clinical Trials Centre Cologne (ZKS Köln) Cologne Germany; ^8^ University of Genoa and Policlinico San Martino IRCCS Hospital Genoa Italy; ^9^ Department of Medicine, Radboudumc Community for Infectious Diseases and Radboud University Medical Center Nijmegen the Netherlands; ^10^ Department of Intensive Care CHU UCL Godinne, UCLouvain, Site Godinne Yvoir Belgium; ^11^ Department of Anesthesiology and Critical Care Medicine Amiens‐Picardie University Hospital Amiens France; ^12^ Medical College of Georgia/Augusta University Augusta Georgia USA; ^13^ Institute of Antibiotics Huashan Hospital, Fudan University Shanghai China; ^14^ Clinical Development Mundipharma Research Limited Cambridge UK; ^15^ University of Alabama at Birmingham Birmingham Alabama USA

**Keywords:** *Candida*, candidaemia, caspofungin, China, efficacy, invasive candidiasis, rezafungin, safety

## Abstract

**Background:**

The efficacy and safety of rezafungin in invasive candidiasis (IC) and candidaemia was demonstrated in the Phase 2 STRIVE and Phase 3 ReSTORE trials, and an additional cohort of patients in ReSTORE from China (ReSTORE China).

**Objectives:**

This post hoc analysis is the first time that combined data from all double‐blind, randomised Phase 2 and 3 rezafungin trials have been evaluated. Outcomes were comprehensively assessed across a large, diverse population.

**Methods:**

Data were assessed from adults with candidaemia/IC in STRIVE (NCT02734862) and ReSTORE (NCT03667690) (including the China extension cohort) who received weekly rezafungin 400/200 mg or daily caspofungin 70/50 mg for ≤ 4 weeks. Efficacy was assessed in the modified intent‐to‐treat population. Endpoints were Day 30 all‐cause mortality (ACM, primary), mycological response at Days 5 and 14 (secondary) and time to negative blood cultures (TTNBC, secondary). Treatment‐emergent adverse events were assessed in the safety population.

**Results:**

Overall, 372 patients were included (rezafungin 179; caspofungin 193). Day‐30 ACM was 20% (33/161) for rezafungin versus 22% (39/178) for caspofungin (weighted difference, −2.4% [95% confidence interval −11.2 to 6.4]). Day‐5 and Day‐14 mycological eradication rates were 73% and 70% for rezafungin versus 65% and 68% for caspofungin, respectively. Median TTNBC was shorter for rezafungin than for caspofungin (23.1 versus 36.7 h, respectively; *p* = 0.0073). Adverse‐event profiles were generally similar across treatments.

**Conclusions:**

This pooled analysis of Phase 2 and Phase 3 data, including the China extension cohort, supports the efficacy and safety of rezafungin. Rezafungin was non‐inferior to caspofungin, with earlier mycological eradication.

## 1 Introduction

Invasive candidiasis (IC) and candidaemia are prevalent conditions associated with high rates of morbidity and mortality [1, 2, 3, 4]. Most infections happen in hospital (~100 cases per 100,000 admissions) and occur commonly in intensive care units (ICUs) [5, 6, 7]. IC and candidaemia have high crude mortality rates of ~40% [1, 7, 8, 9] and the hospital costs associated with these infections present a major economic burden [3, 10, 11].

Echinocandins are an established class of antifungal drugs recommended as first‐line treatment for many forms of IC and candidaemia [12, 13, 14, 15]. Timely diagnosis is crucial to enable prompt initiation of antifungal drug treatment [14, 16], thus maximising the likelihood of early mycological eradication. However, the global management of IC and candidaemia faces challenges due to the emergence of treatment‐resistant non‐albicans *Candida* species [3]. Reports of azole‐resistant *C. parapsilosis* isolates spreading between patients within the ICU environment [17] and the intra‐hospital spread of azole‐resistant *C. glabrata* [18] highlight this emerging healthcare concern. Furthermore, in 2022, the World Health Organisation recognised *Candida* species as critical‐priority ( *C. auris* and *C. albicans* ) and high‐priority ( *C. tropicalis* and *C. parapsilosis* ) pathogens in their Fungal Priority Pathogens List [19, 20]. The increasing prevalence of treatment‐resistant species [3, 21, 22, 23, 24] indicates a need for new antifungals.

Rezafungin is a next‐generation echinocandin approved for the treatment of IC (including candidaemia) in adults in the USA, UK, European Union, Brazil and the Middle East (United Arab Emirates) [25, 26, 27, 28, 29, 30]. Rezafungin has a similar structure and mechanism of action to those of other echinocandins but exhibits improved chemical and metabolic stability and differentiated pharmacokinetic and pharmacodynamic properties, while demonstrating effective tissue penetration at infection sites. These properties permit once‐weekly dosing and high front‐loaded therapeutic drug exposures [31, 32, 33, 34].

The efficacy and safety of rezafungin were demonstrated in two clinical trials with comparable design in patients with candidaemia or IC: the Phase 2 STRIVE trial and the Phase 3 ReSTORE trial [25, 35]. In the ReSTORE trial, rezafungin was non‐inferior to caspofungin for all‐cause mortality (ACM) at Day 30 and global cure at Day 14, with a potential benefit in terms of earlier mycological eradication and shorter time to negative blood culture (TTNBC) [35]. A pooled analysis of STRIVE and ReSTORE supported the findings from the primary analyses [36], with a recent analysis suggesting that rezafungin could be an effective treatment for IC or candidaemia irrespective of baseline *Candida* species (including *C. parapsilosis* and *C. glabrata* ) [37]. An additional cohort of patients from China was recruited to the ReSTORE trial to fulfil regulatory requirements (ReSTORE China), which enabled analysis of rezafungin in a larger, more diverse patient population [38, 39]. Analysis of the results from ReSTORE China, and from a combined analysis of ReSTORE and ReSTORE China, were consistent with those of the primary analysis of ReSTORE [38, 39]. Additionally, population pharmacokinetic analysis found no clinically meaningful difference in rezafungin exposure between the China and non‐China patient cohorts, indicating that dose adjustments are not required in patients from China [39].

Herein, we report the first pooled analysis of data from all Phase 2 and Phase 3 trials evaluating the efficacy and safety of rezafungin, including the STRIVE and ReSTORE trials and the ReSTORE China extension cohort.

## 2 Patients and Methods

### 2.1 Patients and Trial Design

Full methodologies of the double‐blind, multicentre, randomised controlled Phase 2 STRIVE (NCT02734862) and Phase 3 ReSTORE (NCT03667690) trials, including the ReSTORE China extension cohort, have been reported previously [25, 35, 36, 38]. Patients were adults with systemic signs of IC and/or candidaemia, and mycological evidence of infection taken from blood or sterile sampling sites within 96 h before randomisation. Patients were randomised to receive either rezafungin or caspofungin.

This pooled analysis of data from STRIVE, ReSTORE and the ReSTORE China extension cohort examines efficacy and safety in patients who received intravenous rezafungin 400/200 mg once weekly or caspofungin 70/50 mg once daily (The STRIVE study also included patients receiving intravenous rezafungin 400 mg once weekly. Data for these patients were excluded from this analysis, ensuring that all data were derived from the same dosing regimen.). Rezafungin was administered on Day 1 (400 mg) and Day 8 (200 mg), with optional 200‐mg doses given on Day 15 (in both studies) and Day 22 (in ReSTORE and for patients with IC in STRIVE). Caspofungin was given on Day 1 (70 mg), and then daily (50 mg) for at least 3 and up to 21 days (or up to 28 days in ReSTORE and for patients with IC in STRIVE). Those who met step‐down criteria after ≥ 3 days were able to switch to oral step‐down therapy: to placebo from rezafungin and to fluconazole from caspofungin. Those stepping down to fluconazole received an 800‐mg loading dose, followed by 400 mg daily in STRIVE, or 6 mg/kg (up to a daily maximum of 800 mg) in ReSTORE. For both studies, step‐down dosage adjustments were permitted depending on renal function and/or haemodialysis status.

The susceptibility of *Candida* isolates to rezafungin and caspofungin was evaluated at baseline by determining minimum inhibitory concentrations using formal breakpoints published by the Clinical and Laboratory Standards Institute (for rezafungin and caspofungin) and European Committee on Antimicrobial Susceptibility Testing (EUCAST) (for rezafungin) [40]. For caspofungin, neither breakpoints nor epidemiological cut‐off values have been determined by EUCAST.

### 2.2 Endpoints

The primary efficacy endpoint evaluated in this pooled analysis was ACM at Day 30, defined as the proportion of patients who died, irrespective of cause, on or before Day 30, or those with unknown survival status [36]. The exception was patients who were alive at Day 28 or Day 29 but who had unknown survival status at Day 30; these patients were considered alive for the calculation of Day‐30 ACM.

Secondary and exploratory efficacy endpoints comprised mycological response at Days 5 and 14, and TTNBC [36]. Mycological eradication was defined, for patients with a positive blood culture at baseline, as a negative blood culture at Day 5 or 14 with no subsequent positive culture. In patients with a positive culture at baseline from a normally sterile site other than blood, mycological eradication was either documented (negative culture from the same site on or prior to Day 5 or 14) or presumed (by assessment of clinical and radiological cure [if applicable] if a specimen was not available). Mycological eradication also required patients to be alive, have had no change in their antifungal therapy and have not been lost to follow‐up. TTNBC was defined as the time from the first dose of study drug to the first *Candida*‐negative blood culture, without subsequent positive culture. TTNBC was assessed in patients with a positive culture before randomisation and in those with positive culture close to randomisation (patients with either [25] a positive culture from blood drawn between 12 h before and 72 h after randomisation or [35] a positive culture from another normally sterile site sampled between 48 h before and 72 h after randomisation). The proportions of patients with negative blood cultures at 24 h and 48 h after the first dose of study drug were also calculated. Subgroup analyses were conducted for the efficacy endpoints (excluding TTNBC), according to a range of patient‐related parameters and by *Candida* species.

An additional exploratory endpoint was the total length of ICU stay, defined as the total number of days in the ICU during the study period, including re‐admissions. Detailed description of the length‐of‐stay analysis methodology has been reported previously [41].

Safety was assessed via monitoring treatment‐emergent adverse events (TEAEs; adverse events occurring during or after study drug administration until the follow‐up visit), serious adverse events (SAEs), clinical laboratory evaluations and vital signs [35, 36]. TEAEs were coded using Medical Dictionary for Regulatory Activities version 23.0. In STRIVE, adverse events were graded as mild, moderate or severe. In ReSTORE, severity grading was determined using the National Cancer Institute Common Terminology Criteria for Adverse Events version 5.0. In this pooled analysis, severity was graded as mild (defined as mild or Grade 1), moderate (moderate or Grade 2) or severe (severe or at least Grade 3).

### 2.3 Statistical Analyses

Sample‐size calculations for individual studies were reported previously [25, 35]. Here, efficacy was assessed in the modified intent‐to‐treat (mITT) population, comprising patients with a documented *Candida* infection ≤ 96 h before randomisation who received at least one dose of study drug [36]. The total length of ICU stay was assessed in patients from the mITT population who were in the ICU at randomisation. Safety was evaluated in the safety population, comprising all patients who received at least one dose of study drug.

For Day‐30 ACM, two‐sided 95% confidence intervals (CIs) were calculated for the weighted treatment difference (weighted by study and study part [for the two‐part STRIVE trial]) using stratified Miettinen–Nurminen methodology [36]. Rezafungin was considered non‐inferior to caspofungin for Day‐30 ACM if the upper limit of the 95% CI was below 20% (i.e., a 20% non‐inferiority margin, selected in accordance with US Food and Drug Administration guidelines). Weighted treatment differences and corresponding 95% CIs were also calculated for mycological response at Days 5 and 14 using the same methodology. Kaplan–Meier methods were used to analyse TTNBC; patients were censored if they received an alternative antifungal to the study drug, died or were lost to follow‐up before having a negative blood culture. Statistical significance was determined using a stratified log‐rank test. Safety endpoints were summarised using descriptive statistics.

### 2.4 Ethics Statement

The authors confirm that the ethical policies of the journal, as noted on the journal's author guidelines page, have been adhered to and the appropriate ethical review committee approval has been received. Trials were approved by local ethics committees or institutional review boards and all patients provided written informed consent [25, 35].

## 3 Results

### 3.1 Patients

The pooled analysis population comprised 372 patients (179 for rezafungin and 193 for caspofungin). Patient disposition and the contributions of each primary study to the analysis populations are summarised in Figure S1. The mITT population consisted of 339 patients (161 in the rezafungin group and 178 in the caspofungin group), while the safety population comprised 173 and 191 patients in the rezafungin and caspofungin groups, respectively.

Patient demographics and baseline characteristics were similar between treatment groups (Tables 1, 2), although there was a higher proportion of male patients in the rezafungin group (67% [108/161]), compared with the caspofungin group (57% [102/178]). Around three‐quarters of patients in each treatment group had candidaemia only. The proportion of patients from China was similar between the two groups (rezafungin: 19% [30/161]; caspofungin: 16% [29/178]).

**TABLE 1:** Baseline characteristics (mITT population).

Characteristic	Rezafungin 400/200 mg (*n* = 161)	Caspofungin 70/50 mg (*n* = 178)
Median age, years (range)	59 (18–91)	62 (19–93)
Age category, *n* (%)		
< 65 years	95 (59)	107 (60)
≥ 65 years	66 (41)	71 (40)
Sex, *n* (%)		
Male	108 (67)	102 (57)
Female	53 (33)	76 (43)
Race, *n* (%)		
White	95 (59)	106 (60)
Asian	46 (29)	57 (32)
Black or African American	11 (7)	8 (4)
American Indian or Alaska Native	1 (< 1)	1 (< 1)
Other or not reported	8 (5)	6 (3)
Geographical region, *n* (%)		
Europe and Israel	67 (42)	76 (43)
North and South America	43 (27)	46 (26)
China and Taiwan	30 (19)	29 (16)
Asia‐Pacific (excluding China and Taiwan)	21 (13)	27 (15)
Final diagnosis, *n* (%)		
Candidemia only	120 (75)	136 (76)
IC^a^	41 (25)	42 (24)
Modified APACHE II score, *n/N* (%)^b^		
≥ 20	24/159 (15)	30/175 (17)
< 20	135/159 (85)	145/175 (83)
Mean body mass index, kg/m^2^ (SD)^c^	25.3 (7.6)	24.7 (5.9)
ANC, *n/N* (%)		
< 500 cells/μL	9/157 (6)	10/174 (6)
≥ 500 cells/μL	148/157 (94)	164/174 (94)
Mean estimated creatinine clearance, mL/min (SD)^c^	87.3 (93.5)	85.7 (60.6)
Renal impairment based on creatinine clearance, *n/N* (%)^c^		
≥ 60 mL/min (normal/mild)	84/144 (58)	99/165 (60)
< 60 mL/min (moderate/severe)	60/144 (42)	66/165 (40)
Child–Pugh score category, *n* (%)		
< 7	0	1 (< 1)
≥ 7	4 (2)	10 (6)
No history of liver disease (or not calculated), *n* (%)	157 (98)	167 (94)
Patients in ICU at randomisation, *n* (%)	55 (34)	77 (43)

*Note:* Percentages do not always add up to 100% owing to rounding.

Abbreviations: ANC, absolute neutrophil count; APACHE, Acute Physiology and Chronic Health Evaluation; GCS, Glasgow Coma Score; IC, invasive candidiasis; ICU, intensive care unit; mITT, modified intent‐to‐treat; SD, standard deviation.

^a^ Final diagnosis of IC was determined based on the radiological and/or tissue/fluid culture assessment through Day 14 for ReSTORE and was the same as the original diagnosis for STRIVE.

^b^ Combined APACHE II and GCS, calculated as APACHE II + (15 minus GCS).

^c^ Reported for patients with available data. Body mass index: *n* = 149/161 and *n* = 167/178; estimated creatinine clearance: *n* = 144/161 and *n* = 165/178.

Most patients in both treatment groups had baseline modified Acute Physiology and Chronic Health Evaluation II scores < 20 (85% [135/159] in the rezafungin group and 83% [145/175] in the caspofungin group). Additionally, 94% of patients in each group had an absolute neutrophil count ≥ 500 cells/μL at baseline (148/157 and 164/174 for rezafungin and caspofungin, respectively).

A similar distribution of *Candida* species was observed across treatment groups at baseline (Table S1). *C. albicans* was the most frequently isolated species, present in 40% of rezafungin‐treated and 43% of caspofungin‐treated patients, followed by *C. glabrata* (25% and 21%, respectively), *C. tropicalis* (21% and 16%, respectively) and *C. parapsilosis* (11% and 19%, respectively). For each *Candida* species, all isolates tested at baseline were susceptible to rezafungin and caspofungin.

### 3.2 Efficacy in the mITT Population

Based on the prespecified 20% non‐inferiority margin, rezafungin was non‐inferior to caspofungin with respect to Day‐30 ACM (Table 2). Overall Day‐30 ACM rates were 20% (33/161) for rezafungin and 22% (39/178) for caspofungin, with a weighted treatment difference (95% CI) of −2.4% (−11.2 to 6.4). For patients with candidaemia only, Day‐30 ACM rates were 24% (29/120) for rezafungin and 26% (35/136) for caspofungin, with a weighted treatment difference (95% CI) of −2.0% (−12.6 to 8.6). For patients with IC, Day‐30 ACM rates were 10% (4/41) and 10% (4/42) with rezafungin and caspofungin, respectively, for a weighted treatment difference (95% CI) of 0.7% (−14.9 to 16.2).

**TABLE 2:** All‐cause mortality at Day 30 (mITT population).

Endpoint	Rezafungin 400/200 mg (*n* = 161)	Caspofungin 70/50 mg (*n* = 178)	Weighted treatment difference for rezafungin – caspofungin, % (95% CI)^a^
All‐cause mortality at Day 30, *n* (%)			
Died	33 (20)	39 (22)	−2.4 (−11.2 to 6.4)
Known to have died	24 (15)	31 (17)	
Unknown survival status	9 (6)	8 (4)	
All‐cause mortality at Day 30 by diagnosis, *n*/*N* (%)			
Candidaemia only	29/120 (24)	35/136 (26)	−2.0 (−12.6 to 8.6)
IC	4/41 (10)	4/42 (10)	0.7 (−14.9 to 16.2)

Abbreviations: CI, confidence interval; IC, invasive candidiasis; mITT, modified intent‐to‐treat.

^a^ Two‐sided 95% CI for the weighted difference (%), rezafungin – caspofungin group, adjusted for study and study part (latter for STRIVE).

Mycological response at Day 5 and Day 14 is summarised in Table 3. The Day‐5 mycological eradication rate was 73% (117/161) with rezafungin and 65% (116/178) with caspofungin (weighted treatment difference [95% CI], 8.6% [−1.1 to 18.3]). There were treatment differences in favour of rezafungin at Day 5 in patients with candidaemia only (weighted treatment difference [95% CI], 12.2% [1.7 to 22.8]) and in patients with a positive *Candida* blood culture close to randomisation (i.e., patients with either [25] a positive culture from blood drawn between 12 h before and 72 h after randomisation or [35] a positive culture from another normally sterile site sampled between 48 h before and 72 h after randomisation; weighted treatment difference [95% CI], 15.5% [0.3 to 30.6]). Mycological eradication rates were similar at Day 14: 70% (113/161) and 68% (121/178) for rezafungin and caspofungin, respectively (weighted treatment difference [95% CI], 3.0% [−6.8 to 12.8]). A numerically larger, but non‐significant treatment difference was observed at Day 14 in patients with a positive *Candida* blood culture close to randomisation (weighted treatment difference [95% CI], 10.4% [−4.7 to 25.5]).

**TABLE 3:** Mycological response at day 5 and day 14 (mITT population).

Mycological response, *n* (%)	Rezafungin 400/200 mg (*n* = 161)	Caspofungin 70/50 mg (*n* = 178)	Weighted treatment difference for rezafungin – caspofungin, % (95% CI)^a^
Day 5			
Eradication	117 (73)	116 (65)	8.6 (−1.1 to 18.3)
Failure or indeterminate	44 (27)	62 (35)	
Failure	37 (23)	52 (29)	
Indeterminate	7 (4)	10 (6)	
Eradication in patients with candidaemia only, *n*/*N* (%)	95/120 (79)	92/136 (68)	12.2 (1.7 to 22.8)
Eradication in patients with a positive *Candida* culture close to randomisation, *n*/*N* (%)^b^	45/63 (71)	47/85 (55)	15.5 (0.3 to 30.6)
Day 14			
Eradication	113 (70)	121 (68)	3.0 (−6.8 to 12.8)
Failure or indeterminate	48 (30)	57 (32)	
Failure	42 (26)	53 (30)	
Indeterminate	6 (4)	4 (2)	
Eradication in patients with candidaemia only, *n*/*N* (%)	86/120 (72)	94/136 (69)	2.0 (−9.2 to 13.2)
Eradication in patients with a positive *Candida* culture close to randomisation, *n*/*N* (%)^b^	44/63 (70)	51/85 (60)	10.4 (−4.7 to 25.5)

Abbreviations: CI, confidence interval; mITT, modified intent‐to‐treat.

^a^ Two‐sided 95% CI for the weighted difference (%), rezafungin – caspofungin group, adjusted for study and study part (latter for STRIVE).

^b^ Positive *Candida* culture close to randomisation was defined as patients with a positive culture from blood drawn within 12 h prior to or within 72 h after randomisation, or a positive culture from another normally sterile site obtained within 48 h prior to or within 72 h after randomisation.

**FIGURE 1:**
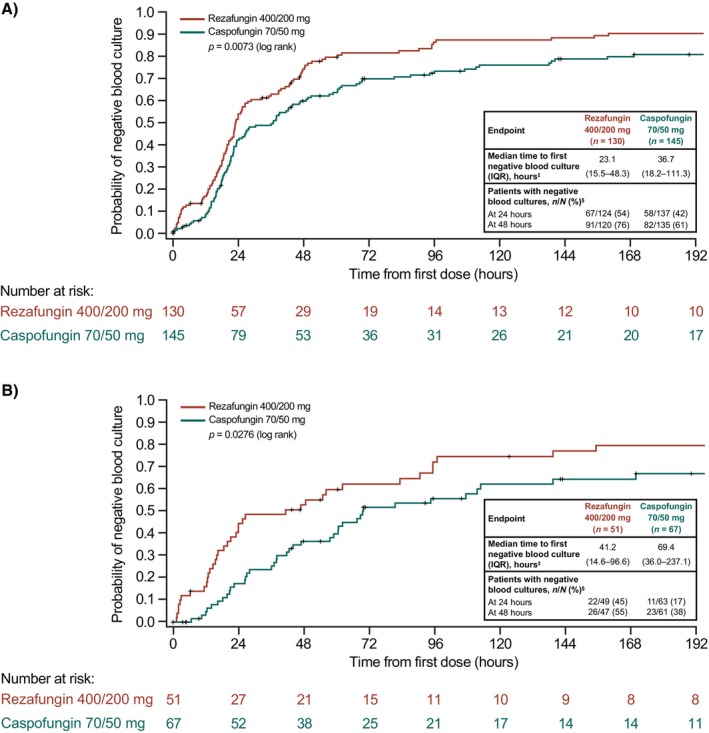
Time to negative blood culture (mITT population). (A) Patients with positive blood culture before randomisation. (B) Patients with positive blood culture close to randomisation. ^†^Patients with a culture from blood drawn within 12 h prior to or within 72 h after randomisation, or a culture from another normally sterile site obtained within 48 h prior to or within 72 h after randomisation. ^‡^Time to first negative blood culture, without subsequent positive culture, from a sample drawn following the first dose of study drug. Patients were censored (indicated with tick marks) if they received an alternative antifungal (i.e., other than study drug) for the treatment of the candidaemia, died or were lost to follow‐up prior to having the negative blood culture. Time to first negative blood culture and percentages are based on Kaplan–Meier estimates. *p* value is from a log‐rank test stratified by study and study part (latter for STRIVE). ^§^Patients censored prior to 24 and 48 h were excluded from the denominator for 24 h and 48 h, respectively. IQR, interquartile range; mITT, modified intent‐to‐treat.

The proportions of patients with negative blood cultures at 24 and 48 h were higher for rezafungin than for caspofungin (Figure 1). In addition, a numerically shorter median TTNBC was observed in patients receiving rezafungin when compared with caspofungin. In patients with positive blood culture before randomisation, 54% (67/124) and 42% (58/137) of patients in the rezafungin and caspofungin groups, respectively, had a negative blood culture at 24 h. At 48 h, this was 76% (91/120) and 61% (82/135), respectively (Figure 1A). In patients with positive blood culture close to randomisation, 45% (22/49) and 17% (11/63) of patients in the rezafungin and caspofungin groups, respectively, had a negative blood culture at 24 h. At 48 h, this was 55% (26/47) and 38% (23/61), respectively (Figure 1B).

Median TTNBC was numerically shorter overall in the rezafungin group than in the caspofungin group. In patients with positive blood culture before randomisation, median (interquartile range [IQR]) TTNBC was 23.1 (15.5–48.3) and 36.7 (18.2–111.3) hours for rezafungin and caspofungin, respectively (*p* = 0.0073) (Figure 1A). In patients with positive blood culture close to randomisation, median (IQR) TTNBC was 41.2 (14.6–96.6) and 69.4 (36.0–237.1) hours for rezafungin and caspofungin, respectively (*p* = 0.0276) (Figure 1B).

### 3.3 Efficacy by *Candida* Species

Day‐30 ACM analyses stratified by baseline *Candida* species were limited by small numbers of patients per subgroup (Table S2). For the largest subgroup, *C. albicans* , rates were broadly comparable between rezafungin (25% [16/64]) and caspofungin (22% [17/76]). Day‐5 and Day‐14 mycological eradication rates for the four most prevalent species in this pooled analysis were higher for rezafungin than caspofungin, but differences did not reach statistical significance (Table 4).

**TABLE 4:** Mycological eradication at Day 5 and Day 14 by baseline *Candida* species (mITT population).

Mycological eradication rate, *n*/*N* (%)^a^	Rezafungin 400/200 mg	Caspofungin 70/50 mg	Treatment difference for rezafungin – caspofungin, % (95% CI)^b^
Day 5			
*C. albicans*	47/64 (73)	50/76 (66)	7.6 (−7.9 to 22.5)
*C. glabrata*	30/40 (75)	22/37 (59)	15.5 (−5.5 to 35.7)
*C. tropicalis*	26/34 (76)	17/28 (61)	15.8 (−7.4 to 38.2)
*C. parapsilosis*	14/18 (78)	22/33 (67)	11.1 (−16.3 to 34.1)
*C. krusei*	2/5 (40)	2/3 (67)	—
Day 14			
*C. albicans*	43/64 (67)	50/76 (66)	1.4 (−14.4 to 16.9)
*C. glabrata*	33/40 (83)	24/37 (65)	17.6 (−2.1 to 36.8)
*C. tropicalis*	24/34 (71)	18/28 (64)	6.3 (−16.9 to 29.5)
*C. parapsilosis*	14/18 (78)	23/33 (70)	8.1 (−19.1 to 30.9)
*C. krusei*	2/5 (40)	3/3 (100)	—

Abbreviations: CI, confidence interval; mITT, modified intent‐to‐treat.

^a^ The numerator (*n*) is the number of patients with the corresponding *Candida* species who had mycological eradication. The denominator (*N*) is the number of patients with the corresponding *Candida* species at baseline. Only *Candida* species for which there were at least five cases in either group are shown.

^b^ Two‐sided 95% CI for the difference (%), rezafungin – caspofungin group, unadjusted. 95% CIs were only determined for *Candida* species for which there were at least 10 cases in either group.

### 3.4 Efficacy in Other Subgroups and Special Populations

Exploratory subgroup analyses for Day‐30 ACM and mycological eradication rates are summarised in Figures S2 and S3, respectively.

#### 3.4.1 Older Patients

Among patients ≥ 65 years old, Day‐30 ACM rates were 18% (12/66) for rezafungin and 34% (24/71) for caspofungin, with a weighted treatment difference (95% CI) of −16.3% (−30.6 to −1.9). In these patients, weighted treatment differences (95% CI) for mycological eradication were 16.4% (1.2 to 31.5) at Day 5 and 18.6% (3.7 to 33.4) at Day 14.

#### 3.4.2 Patients With Obesity

Among patients with a body mass index > 30 kg/m^2^, Day‐30 ACM rates were 23% (7/30) for rezafungin and 18% (4/22) for caspofungin, with a weighted treatment difference (95% CI) of 0.2% (−23.5 to 23.8). In this subgroup of patients, weighted treatment differences (95% CI) for mycological eradication were 1.3% (−24.1 to 26.7) at Day 5 and −6.9% (−31.6 to 17.8) at Day 14.

#### 3.4.3 Patients With Renal Insufficiency

In patients with an estimated creatinine clearance rate of < 60 mL/min (indicating moderate‐to‐severe renal insufficiency), Day‐30 ACM rates were 17% (10/60) for rezafungin and 30% (20/66) for caspofungin, with a weighted treatment difference (95% CI) of −15.0% (−29.5 to −0.6). Weighted treatment differences (95% CI) for mycological eradication at Day 5 and Day 14 in these patients were 10.0% (−6.0 to 25.9) and 13.7% (−2.2 to 29.5), respectively.

#### 3.4.4 Patients With Hepatic Impairment

The number of patients with hepatic impairment (as indicated by a Child–Pugh score of ≥ 7) was too small (rezafungin, *n* = 4; caspofungin, *n* = 10) to permit meaningful analyses of treatment efficacy in this subgroup.

#### 3.4.5 Patients in the ICU at Randomisation

In patients in the ICU at randomisation, the proportion of patients with an APACHE II score of ≥ 20 was similar between the rezafungin (34.0% [18/53]) and caspofungin (29.9% [23/77]) groups. Day‐30 ACM rates for patients in the ICU were 38% (21/55) for rezafungin and 30% (23/77) for caspofungin, with a weighted treatment difference (95% CI) of 6.5% (−9.1 to 22.9). In this subgroup, mycological eradication rates were 76% (42/55) for rezafungin and 60% (46/77) for caspofungin at Day 5 and 69% (38/55) and 64% (49/77), respectively, at Day 14. Weighted treatment differences (95% CI) for mycological eradication at Day 5 and Day 14 in these patients were 17.1% (0.5 to 32.3) and 6.4% (−10.4 to 22.0), respectively.

In this subgroup, 53% (23/43) and 36% (21/59) of patients in the rezafungin and caspofungin groups, respectively, had a negative blood culture at 24 h, with a treatment difference (95% CI) of 17.9% (−1.7 to 36.3). At 48 h, these proportions were 78% (32/41) and 50% (29/58), respectively, with a treatment difference (95% CI) of 28.0% (8.8 to 44.8). Median (IQR) TTNBC among patients in the ICU at randomisation was 22.9 (14.3–47.6) hours with rezafungin and 48.9 (17.8–211.3) hours with caspofungin (*p* = 0.003).

Median (range) length of stay in the ICU across all admissions in this subpopulation was 13 (2–48) days in the rezafungin group and 17 (1–61) days in the caspofungin group.

### 3.5 Safety

Rezafungin and caspofungin demonstrated similar safety profiles (Table 5). TEAEs were reported in 92% (159/173) and 85% (162/191) of patients during rezafungin and caspofungin treatment, respectively (Table 5). Drug‐related TEAEs occurred in 16% (27/173) of patients in the rezafungin group and in 13% (24/191) in the caspofungin group.

**TABLE 5:** Treatment‐emergent adverse events (safety population).

Patients with at least one TEAE, *n* (%)	Rezafungin	Caspofungin
	400/200 mg	70/50 mg
	(*n* = 173)	(*n* = 191)
Any TEAE	159 (92)	162 (85)
Any drug‐related TEAE	27 (16)	24 (13)
TEAE at or above Grade 3	88 (51)	104 (54)
SAE	92 (53)	95 (50)
SAE leading to death	39 (23)	50 (26)
Drug‐related SAE	3 (2)	5 (3)
Drug‐related SAE leading to death	0	0
TEAE leading to:		
Interruption of study drug	3 (2)	4 (2)
Discontinuation of study drug	15 (9)	16 (8)
Study discontinuation	26 (15)	34 (18)
TEAEs occurring in at least 5% of patients in either arm		
Hypokalaemia	26 (15)	22 (12)
Diarrhoea	20 (12)	18 (9)
Anaemia	19 (11)	15 (8)
Pyrexia	19 (11)	13 (7)
Vomiting	15 (9)	8 (4)
Pneumonia	14 (8)	11 (6)
Nausea	14 (8)	9 (5)
Septic shock	13 (8)	15 (8)
Abdominal pain	13 (8)	9 (5)
Hypomagnesaemia	12 (7)	7 (4)
Sepsis	11 (6)	9 (5)
Insomnia	9 (5)	4 (2)
Acute kidney injury	7 (4)	12 (6)
Hypotension	7 (4)	11 (6)
Urinary tract infection	6 (3)	10 (5)
Respiratory failure	4 (2)	10 (5)
Pleural effusion	3 (2)	11 (6)

Abbreviations: SAE, serious adverse event; TEAE, treatment‐emergent adverse event.

The most common TEAEs experienced by patients were hypokalaemia (rezafungin 15% [26/173] vs. caspofungin 12% [22/191]), diarrhoea (12% [20/173] vs. 9% [18/191]), anaemia (11% [19/173] vs. 8% [15/191]) and pyrexia (11% [19/173] vs. 7% [13/191]) (Table 5). TEAEs of Grade 3 or higher occurred in 51% (88/173) of patients receiving rezafungin and 54% (104/191) of patients receiving caspofungin, while TEAEs resulting in study drug discontinuation occurred in 9% (15/173) and 8% (16/191) of patients in the rezafungin and caspofungin groups, respectively.

SAEs occurred in 53% (92/173) and 50% (95/191) of patients in the rezafungin and caspofungin groups, respectively. SAEs resulting in death occurred in 23% (39/173) of patients in the rezafungin group and 26% (50/191) of patients in the caspofungin group. These were not considered to be related to study drug. The most common SAE was septic shock (in 5% [9/173] and 7% [13/191] of patients in rezafungin and caspofungin treatment groups, respectively) (Table S3). Drug‐related SAEs occurred in 2% (3/173) and 3% (5/191) of patients in the rezafungin and caspofungin groups, respectively (Table 5).

## 4 Discussion

This is the first pooled analysis of data from all Phase 2 (STRIVE) and Phase 3 (ReSTORE) rezafungin trials, incorporating recently available data from the ReSTORE China extension cohort. It represents the largest, most diverse patient population reported to date. In line with earlier analyses, rezafungin was non‐inferior to caspofungin in terms of efficacy, with a comparable safety profile.

The non‐inferiority of rezafungin to caspofungin was confirmed with respect to Day‐30 ACM rate (20% vs. 22% respectively; weighted treatment difference [95% CI], −2.4% [−11.2 to 6.4]) and rates were similar between treatment groups regardless of diagnosis. Similarly, mycological eradication rates at Days 5 and 14 were comparable between treatments. A higher proportion of patients had negative blood cultures at 24 and 48 h with rezafungin than caspofungin, and a shorter TTNBC was observed with rezafungin compared with caspofungin. Safety and tolerability were comparable between rezafungin and caspofungin, and reported TEAEs were consistent with the known safety profiles of both agents [25, 26, 28, 35, 42].

In addition to supporting the findings of earlier reports [25, 35], the present exploratory subgroup analyses suggested some potential treatment differences among certain patient subpopulations, generally in favour of rezafungin. Day‐5 mycological eradication rates in patients with candidaemia only and in patients with a positive *Candida* blood culture close to randomisation significantly favoured rezafungin over caspofungin. Among older patients, Day‐30 ACM rates were significantly lower and mycological eradication rates at both Day 5 and Day 14 were significantly higher with rezafungin versus caspofungin. Similarly, Day‐30 ACM rates in patients with renal insufficiency were significantly lower in the rezafungin group versus the caspofungin group.

This extensive analysis also indicated a potential early treatment benefit for rezafungin. Mycological eradication rates at Day 5 were numerically higher with rezafungin (73% [117/161]) versus caspofungin (65% [116/178]; weighted treatment difference [95% CI], 8.6% [−1.1 to 18.3]). A numerically shorter TTNBC was also seen with rezafungin compared with caspofungin. Similar observations were seen in patients with a positive *Candida* culture close to randomisation (both endpoints) and in patients with candidaemia only (mycological eradication rate at Day 5). In accordance with a previous pooled analysis of the STRIVE and ReSTORE data [43], similar findings were also observed among patients in the ICU at randomisation. Such rapid infection clearance may be important for this critically ill population who appear to be especially vulnerable to resistance development and negative outcomes with inadequate initial dosing [44, 45]. Notably, the larger dataset available for this pooled analysis may have improved the statistical power to detect treatment differences; however, further investigations into the potential early treatment benefit with rezafungin are required.

The numerically shorter TTNBC observed with rezafungin compared with caspofungin suggests a potential to reduce the risk of *Candida* dissemination and deep organ seeding; however, this hypothesis remains speculative and requires further investigation. A recent post hoc analysis of data from ReSTORE and ReSTORE China showed that early mycological eradication with rezafungin was associated with a numerically lower mortality rate and a numerically shorter length of ICU stay compared with caspofungin in patients with candidaemia [46]. The early treatment benefits of rezafungin may be related to its differentiated pharmacokinetic/pharmacodynamic profile, with low clearance and a prolonged half‐life (~133 h) that enables front‐loaded exposure [33, 47]. Potentially, these attributes could also contribute to preventing mutant strain selection. In a mouse model of intra‐abdominal candidiasis, intraperitoneal rezafungin showed more sustained penetration into abscesses than micafungin at “humanised therapeutic doses” [34]. Unlike micafungin, mean drug concentrations for rezafungin in abscesses were above the mutant prevention concentrations for *C. albicans* and *C. glabrata* adopted by the authors. Interestingly, in a hospital‐based study of 112 patients with intra‐abdominal *Candida* infections, 10 had isolates resistant to micafungin and/or anidulafungin; of these, seven had received/were receiving a first‐generation echinocandin (although the impact of dosing and compliance are unclear as these were not reported) [48]. These findings are generally consistent with those from a study of 23 critically ill patients with suspected *Candida* peritonitis: median steady‐state levels of micafungin, anidulafungin, or caspofungin in peritoneal fluid were below the mutant prevention concentrations adopted by the authors [49]. Beyond mutant selection, clinicians also need to consider tailoring therapy for biofilm‐associated infections. In vitro research shows that the antibiofilm activity of multiple antifungals, including rezafungin and caspofungin, varies against biofilms of *Candida* species, especially *C. auris* and *C. parapsilosis* [50]. Further research in this setting would be valuable.

A key strength of the analysis is the large and diverse dataset. Previous pooled analyses of STRIVE and ReSTORE included an analysis population of 294 patients [36, 37]. The inclusion of the ReSTORE China cohort in the present analysis increases the analysis population to 339 patients. The additional patients from China were distributed equally between treatment groups and the baseline characteristics of patients were also broadly similar between this pooled population and the ReSTORE China cohort [39]. There was, however, a higher proportion of males in the rezafungin group versus the caspofungin group (67% vs. 57%, respectively). Importantly, population pharmacokinetic analysis suggests that this imbalance is unlikely to have impacted findings [51].

A potential study limitation is the exclusion of paediatric patients and individuals with specific forms of IC (e.g., osteomyelitis, endocarditis, central nervous system infection), which typically require prolonged antifungal therapy or occur in sites where there is poor drug penetration [35]. The applicability of these findings to those patient subsets therefore warrants further investigation, especially as case reports/series suggest rezafungin may be clinically effective in individuals with certain complex forms of IC [52, 53, 54, 55, 56, 57, 58, 59, 60, 61]. Some methodological differences between STRIVE and ReSTORE were also apparent (as noted earlier); many of these were discussed in previous pooled analyses [36, 37]. Key differences include geographical distribution, dosing regimens in STRIVE, fluconazole step‐down dosing, minimum inhibitory concentration testing protocols, AE grading and definitions of global cure and clinical cure. Differences in cure definitions and assessment timings led to the exclusion of these outcomes in this pooled analysis; otherwise, the statistical approach employed accounted for important methodological differences, with data stratified (by study and study part), excluded, and pooled where appropriate for consistent analysis.

## 5 Conclusions

This pooled analysis of Phase 2 and Phase 3 clinical trial data, including an extension cohort from China, confirms the efficacy and safety of rezafungin for the treatment of candidaemia and IC in adults, and demonstrates its non‐inferiority to caspofungin for ACM at Day 30. Rezafungin was also associated with earlier mycological eradication compared with caspofungin, particularly among patients with a positive *Candida* culture close to randomisation, those with candidaemia only and in patients admitted to the ICU at randomisation. These findings further reinforce the potential clinical benefits of rezafungin in this high‐risk population.

## Author Contributions

**Alex Soriano:** investigation, validation, visualization, writing – review and editing. **George R. Thompson III:** investigation, validation, visualization, writing – review and editing. **Oliver A. Cornely:** conceptualization, formal analysis, methodology, validation, visualization, writing – review and editing. **Matteo Bassetti:** supervision, validation, writing – review and editing. **Bart Jan Kullberg:** conceptualization, formal analysis, investigation, methodology, validation, visualization, writing – review and editing. **Patrick M. Honoré:** investigation, validation, visualization, writing – original draft, writing – review and editing. **Hervé Dupont:** validation, visualization, writing – original draft, writing – review and editing. **Jose A. Vazquez:** conceptualization, investigation, visualization, writing – original draft, writing – review and editing. **Haihui Huang:** investigation, resources, validation. **Yingyuan Zhang:** investigation, resources, validation, writing – review and editing. **Sara Dickerson:** funding acquisition, resources, validation, visualization, writing – review and editing. **Laura Cox:** formal analysis, funding acquisition, project administration, resources, supervision, validation, visualization, writing – review and editing. **Peter G. Pappas:** investigation, visualization, writing – review and editing.

## Funding

The STRIVE trial was funded by Cidara Therapeutics (San Diego, CA, USA). The ReSTORE trial was co‐funded by Cidara Therapeutics (San Diego, CA, USA) and Mundipharma Research Limited (Cambridge, UK). Cidara Therapeutics was involved in the study design, study conduct, data collection, data analysis and reporting of the trial. Mundipharma Research Limited was involved in the data analysis and reporting of the trial. Funding for medical writing support for this article was provided by Mundipharma Research Limited.

## Conflicts of Interest

A.S. has received fees for lectures from Pfizer, Menarini, MSD, Angelini, Gilead and Shionogi; consulting fees from Pfizer, MSD, Angelini, Shionogi, Gilead and Menarini; support for attending meetings from Pfizer; and grants from Gilead and Pfizer. G.R.T. reports grants or contracts from Astellas, Basilea, Cidara, F2G, Immy and Scynexis; consulting fees from Astellas, Basilea, Cidara, Elion, F2G, GSK, Immy and Scynexis; honoraria from GAFFI; and has participated in a DSMB or advisory board for Pfizer. O.A.C. reports institutional grants or contracts from iMi, iHi, DFG, BMFTR, Cidara, DZIF, EU‐DG RTD, F2G, G‐BA, Gilead, MedPace, MSD, Mundipharma, Octapharma, Pfizer, Scynexis; consulting fees from AbbVie, AiCuris, Basilea, Cidara, Elion, Gilead, GSK, IQVIA, Janssen, MedPace, Menarini, Melinta, Mundipharma, Octapharma, Pardes, Pfizer, PSI, Scynexis, Seqirus, Shionogi; speaker and lecture honoraria from Abbott, AbbVie, Hikma, Asahi Kasei, AstraZeneca, Gilead, GSK, Knight, InfectoPharm, Ipsen, Medscape/WebMD, MSD, Moderna, Mundipharma, Noscendo, Paul‐Martini‐Stiftung, Pfizer, Sandoz, Seqirus, Shionogi, Vitis; participation in a DRC, DSMB, DMC, or Advisory Board for AstraZeneca, Cidara, IQVIA, Janssen, MedPace, Melinta, PSI, Pulmocide, Vedanta. M.B. has received remuneration for Advisory Board, Speaker Activities and Research Funds from Advanz, Angelini, Cidara, Gilead, Menarini, MSD, Pfizer, Shionogi and Mundipharma. B.J.K. reports independent data review committee membership for Cidara. P.M.H. reports grants or contracts from Baxter, Cytosorbents and Pfizer; consulting fees from Baxter, Cytosorbents and Pfizer; honoraria from Baxter and Cytosorbents; and support for attending meetings from Mundipharma and Pfizer. H.D. reports consulting fees from Mundipharma, Shionogi and Pfizer; received fees for lectures from Pfizer, Shionogi and Mundipharma; and received support for attending meetings from Pfizer and MSD. J.A.V. has participated in a DSMB or Advisory Board for F2G and Vedata; and reports consulting fees and honoraria from Melinta. H.H. and Y.Z. report no conflicts of interest. S.D. was an employee of Mundipharma Research Ltd. at the time the work was undertaken. L.C. is an employee of Mundipharma Research Ltd. P.G.P. reports research support from Basilea, Melinta and Scynexis; received consulting fees from F2G; and has participated in a DSMB or Advisory Board for Basilea, Melinta and Scynexis.

## Supporting information


**Table S1:** Baseline *Candida* species from baseline blood and sterile‐site cultures (mITT population).
**Table S2:** All‐cause mortality at Day 30 by baseline *Candida* species (mITT population).
**Table S3:** Serious adverse events occurring in at least three patients in either treatment arm (safety population).
**Figure S1:** Patient flow.
**Figure S2:** Forest plot of weighted differences in all‐cause mortality rates at Day 30 between treatments in patient subgroups (mITT population).
**Figure S3:** Forest plot of weighted differences in mycological eradication rates at (A) Day 5 and (B) Day 14 between treatments in patient subgroups (mITT population).

## Data Availability

Access to the respective study protocols and anonymised data can be requested by contacting enquiries@napp.co.uk. Each request will be reviewed by the sponsor for scientific merit.
