# Supplementary material for: Rezafungin Versus Caspofungin in Candidaemia and Invasive Candidiasis: A Post Hoc Pooled Analysis of Phase 2 and Phase 3 Trials
Source: Mycoses. 2026 Jul 16;69(7):e70205. doi: 10.1111/myc.70205 (PMC13373745; doi:10.1111/myc.70205)
Supplement: Supplementary file 1 — Table S1: Baseline Candida species from baseline blood and sterile‐site cultures (mITT population). Table S2: All‐cause mortality at Day 30 by baseline Candida species (mITT population). Table S3: Serious adverse events occurring in at least three patients in either treatment arm (safety population). Figure S1: Patient flow. Figure S2: Forest plot of weighted differences in all‐cause mortality rates at Day 30 between treatments in patient subgroups (mITT population). Figure S3: Forest plot of weighted differences in mycological eradication rates at (A) Day 5 and (B) Day 14 between treatments in patient subgroups (mITT population). [file MYC-69-e70205-s001.pdf]

Supplementary Materials

Supplement to: A. Soriano, G. R. Thompson III, O. A. Cornely, M. Bassetti, B. J. Kullberg, P. M. Honoré, H. Dupont, J. A. Vazquez, H. Huang, Y. Zhang, S. Dickerson, L. Cox, P. G. Pappas. Rezafungin Versus Caspofungin in Candidaemia and Invasive Candidiasis: A Post-hoc Pooled Analysis of Phase 2 and Phase 3 Trials

TABLE S1 Baseline *Candida* species from baseline blood and sterile-site cultures (mITT population).

***Candida* species, *n* (%)**	**Rezafungin 400/200 mg (*n* = 161)**	**Caspofungin 70/50 mg (*n* = 178)**
C. albicans	64 (40)	76 (43)
C. dubliniensis	3 (2)	2 (1)
C. fabianii (Cyberlindnera fabianii)	0	0
C. glabrata (Nakaseomyces glabrata)	40 (25)	37 (21)
C. guilliermondii (Meyerozyma guilliermondii)	2 (1)	1 (<1)
C. guilliermondii var. *membranifaciens (Kodamaea ohmeri)*	0	1 (<1)
C. kefyr (Kluyveromyces marxianus)	0	1 (<1)
C. krusei (Pichia kudriavzevii)	5 (3)	3 (2)
C. lusitaniae (Clavispora lusitaniae)	1 (<1)	1 (<1)
C. metapsilosis	3 (2)	0
C. nivariensis	0	1 (<1)
C. parapsilosis	18 (11)	33 (19)
C. rugosa	0	0
C. tropicalis	34 (21)	28 (16)

Abbreviation: mITT, modified intent-to-treat.

TABLE S2 All-cause mortality at Day 30 by baseline *Candida* species (mITT population).

***Candida* species, *n*/*N*† (%)**	**Rezafungin 400/200 mg (*n* = 161)**	**Caspofungin 70/50 mg (*n* = 178)**	**Treatment difference for rezafungin – caspofungin, % (95% CI)‡**
C. albicans	16/64 (25)	17/76 (22)	2.6 (−11.4 to 17.1)
C. dubliniensis	0/3 (0)	0/2 (0)	–
C. fabianii (Cyberlindnera fabianii)	0	0	–
C. glabrata (Nakaseomyces glabrata)	7/40 (18)	4/37 (11)	6.7 (−9.9 to 23.1)
C. guilliermondii (Meyerozyma guilliermondii)	0/2 (0)	1/1 (100)	–
C. guilliermondii var. membranifaciens (Kodamaea ohmeri)	0	0/1 (0)	–
C. kefyr (Kluyveromyces marxianus)	0	0/1 (0)	–
C. krusei (Pichia kudriavzevii)	1/5 (20)	0/3 (0)	–
C. lusitaniae (Clavispora lusitaniae)	0/1 (0)	0/1 (0)	–
C. metapsilosis	0/3 (0)	0	–
C. nivariensis	0	0/1 (0)	–
C. parapsilosis	1/18 (6)	9/33 (27)	−21.7 (−40.3 to 1.7)
C. rugosa	0	0	–
C. tropicalis	7/34 (21)	10/28 (36)	−15.1 (−37.2 to 7.3)

†Numerator (*n*) is the number of patients with the corresponding *Candida* species who died on or before Day 30, or with unknown survival status. Denominator (*N*) is the number of patients with the corresponding *Candida* species at baseline. ‡Two-sided 95% CI for the difference in all-cause mortality (rezafungin – caspofungin group) was calculated using the unadjusted Miettinen–Nurminen methodology within each subgroup; 95% CIs were determined only for *Candida* species for which there were at least 10 cases in either group. Abbreviations: CI, confidence interval; mITT, modified intent-to-treat.

TABLE S3 Serious adverse events occurring in at least three patients in either treatment arm (safety population).

**Preferred term, *n* (%)**	**Rezafungin 400/200 mg (*n* = 173)**	**Caspofungin 70/50 mg (*n* = 191)**
Septic shock	9 (5)	13 (7)
Multiple organ dysfunction syndrome	6 (3)	5 (3)
Sepsis	5 (3)	7 (4)
Pneumonia	4 (2)	4 (2)
Bacteraemia	4 (2)	2 (1)
Gastrointestinal haemorrhage	4 (2)	0
Cardiac arrest	3 (2)	1 (<1)
Abdominal abscess	2 (1)	3 (2)
Acute kidney injury	2 (1)	3 (2)
Respiratory failure	2 (1)	7 (4)
Acute respiratory failure	1 (<1)	4 (2)
Aspiration	1 (<1)	3 (2)
Klebsiella sepsis	0	3 (2)
Hyperkalaemia	0	3 (2)

FIGURE S1 Patient flow.

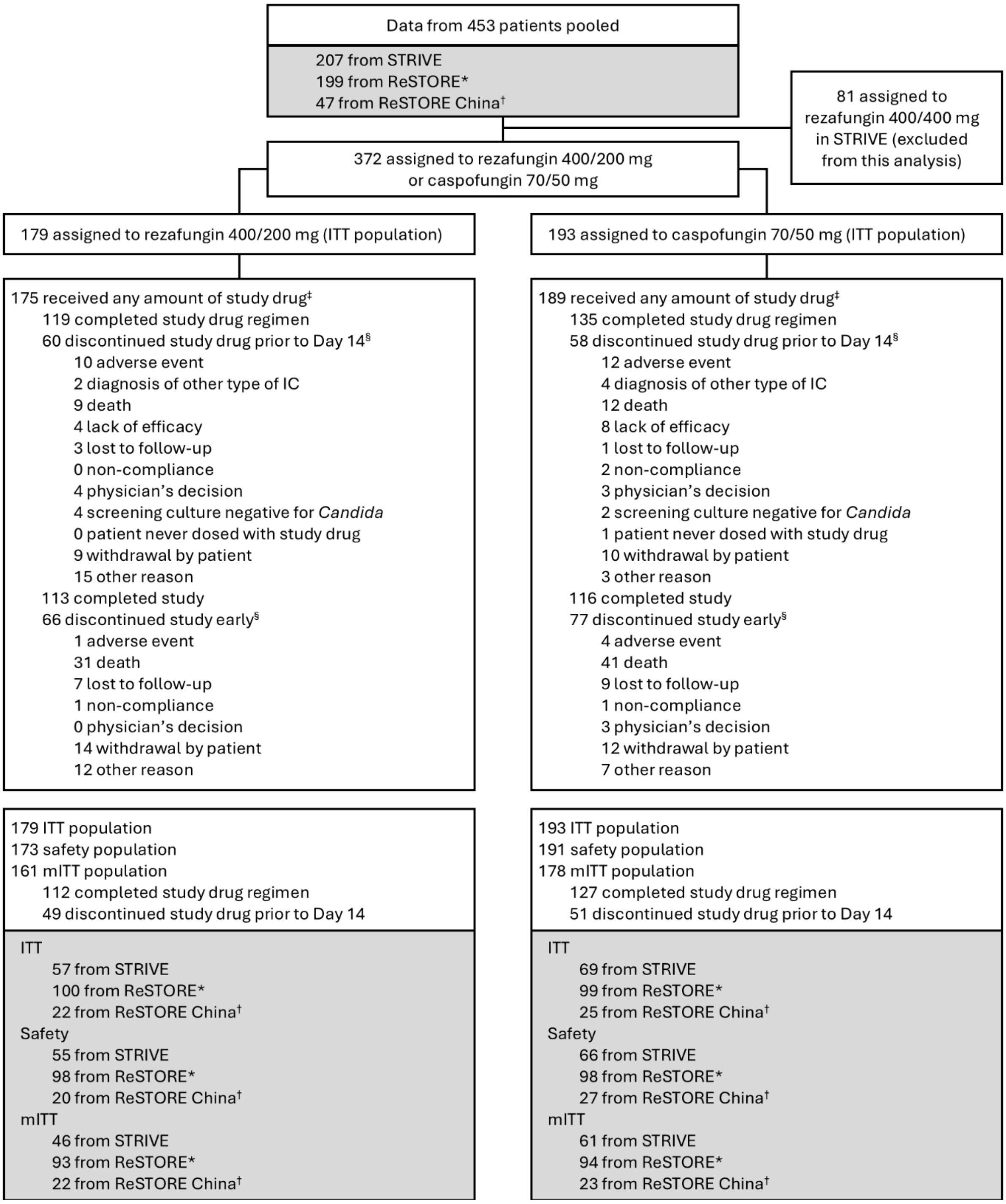

*Includes patients from China enrolled in the original ReSTORE trial (ITT and safety populations, 11 patients; mITT population, 10 patients). †Includes only patients enrolled in the China extension (these patients were not included in previous pooled analyses of STRIVE and ReSTORE [1, 2]). ‡Two patients in STRIVE were randomised to rezafungin 400/200 mg but received caspofungin 70/50 mg. §Only the primary reason for study drug/study discontinuation is shown. Abbreviations: IC, invasive candidiasis; ITT, intent-to-treat; mITT, modified intent-to-treat.

FIGURE S2 Forest plot of weighted differences in all-cause mortality rates at Day 30 between treatments in patient subgroups (mITT population).

Subgroup	Patients who died or with unknown survival status, *n*/*N* (%) Rezafungin 400/200 mg	Caspofungin 70/50 mg	Difference (95% CI) for rezafungin − caspofungin
**Overall**	**33/161 (20)**	**39/178 (22)**	**−2.4 (−11.2 to 6.4)**
**Age**			
18–<65 years	21/95 (22)	15/107 (14)	8.0 (−3.4 to 19.3)
≥65 years	12/66 (18)	24/71 (34)	−16.3 (−30.6 to −1.9)
65–<75 years	7/36 (19)	11/38 (29)	−8.7 (−28.4 to 10.9)
≥75 years	5/30 (17)	13/33 (39)	−20.0 (−40.5 to 0.6)
**Race group**			
White	14/95 (15)	17/106 (16)	−2.8 (−13.3 to 7.6)
Non-White	18/61 (30)	22/68 (32)	−2.5 (−18.2 to 13.2)
**Sex**			
Male	25/108 (23)	21/102 (21)	0.3 (−11.0 to 11.6)
Female	8/53 (15)	18/76 (24)	−7.1 (−21.4 to 7.2)
**Geographical region**			
North/South America	6/43 (14)	4/46 (9)	4.1 (−11.5 to 19.8)
Europe/Israel	10/67 (15)	15/76 (20)	−6.6 (−19.2 to 6.1)
Asia-Pacific (excl. China/Taiwan)	8/21 (38)	10/27 (37)	1.4 (−25.7 to 28.4)
China/Taiwan	9/30 (30)	10/29 (34)	−4.3 (−28.0 to 19.3)
**BMI**			
<25 kg/m^2 (underweight/normal)	16/89 (18)	22/93 (24)	−4.4 (−16.2 to 7.5)
25–30 kg/m^2 (overweight)	7/30 (23)	10/52 (19)	6.0 (−12.7 to 24.7)
>30 kg/m^2 (obese)	7/30 (23)	4/22 (18)	0.2 (−23.5 to 23.8)
**ANC**			
<500 cells/µL	4/9 (44)	3/10 (30)	12.3 (−28.6 to 53.2)
≥500 cells/µL	29/148 (20)	35/164 (21)	−2.5 (−11.6 to 6.6)
**Renal impairment**			
≥60 mL/min (normal/mild)	19/84 (23)	16/99 (16)	6.6 (−5.4 to 18.5)
<60 mL/min (moderate/severe)	10/60 (17)	20/66 (30)	−15.0 (−29.5 to −0.6)
**Modified APACHE II score**			
<10	5/57 (9)	6/57 (11)	−2.7 (−15.2 to 9.8)
10–19	18/78 (23)	20/88 (23)	−1.8 (−14.6 to 11.0)
<20	23/135 (17)	26/145 (18)	−1.3 (−10.6 to 7.9)
≥20	8/24 (33)	13/30 (43)	−8.0 (−31.9 to 15.9)
**Final diagnosis**			
Candidaemia only	29/120 (24)	35/136 (26)	−2.0 (−12.6 to 8.6)
IC	4/41 (10)	4/42 (10)	0.7 (−14.9 to 16.2)
**Positive culture within window**			
Yes	16/63 (25)	21/85 (25)	0.1 (−14.3 to 14.5)
No	17/98 (17)	18/93 (19)	−2.5 (−14.0 to 8.9)
**Patients in ICU at randomisation**	21/55 (38)	23/77 (30)	6.5 (−9.1 to 22.9)

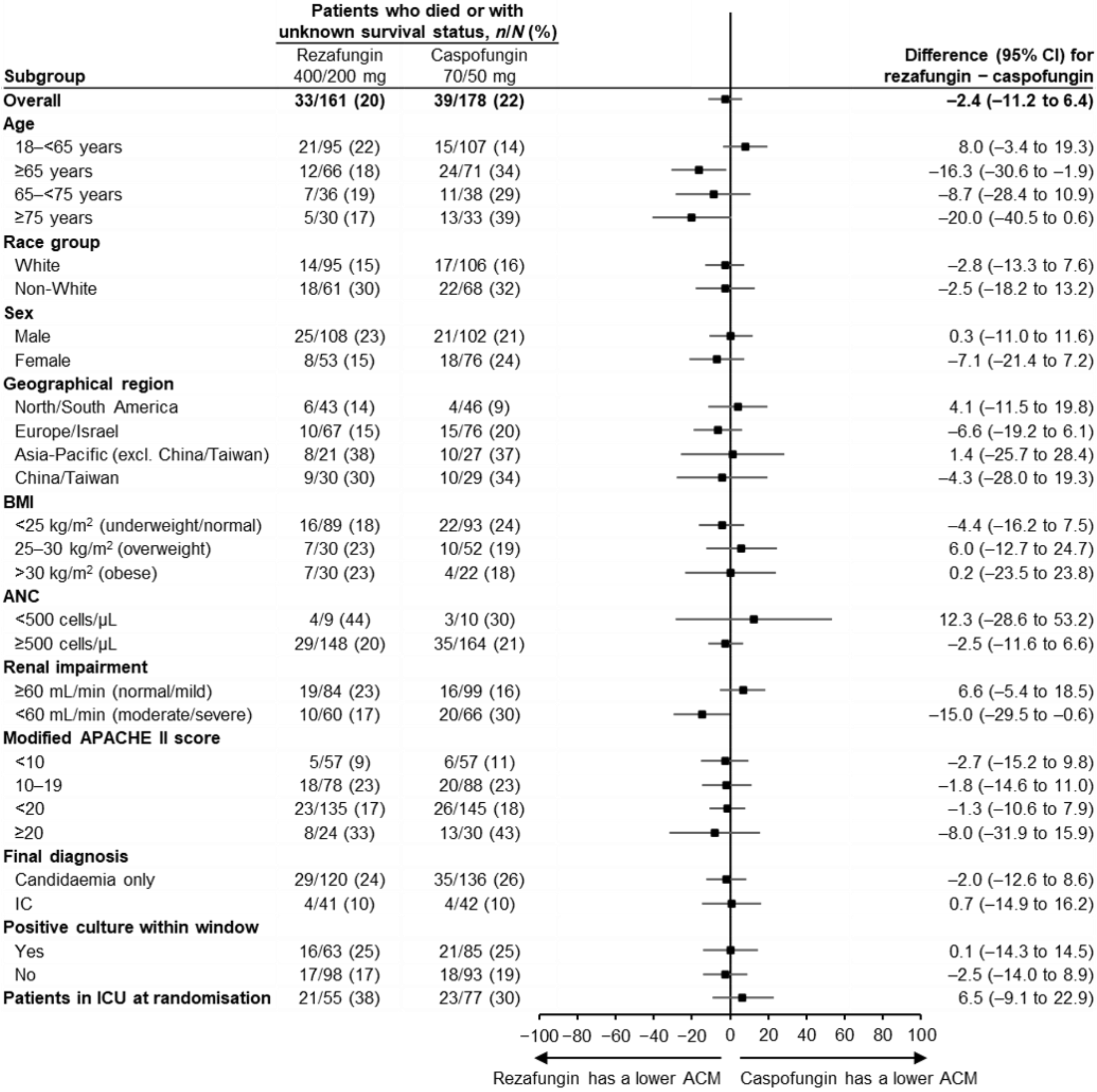

Numbers of patients who died on or before Day 30 include those with unknown survival status. Two-sided 95% CI for the weighted difference in death rates (rezafungin – caspofungin group) is calculated using the stratified (by study and part) Miettinen–Nurminen methodology within each subgroup. Abbreviations: ACM, all-cause mortality; APACHE, Acute Physiology and Chronic Health Evaluation, ANC, absolute neutrophil count; BMI, body mass index; CI, confidence interval; IC, invasive candidiasis; ICU, intensive care unit; mITT, modified intent-to-treat.

FIGURE S3 Forest plot of weighted differences in mycological eradication rates at **A)** Day 5 and **B)** Day 14 between treatments in patient subgroups (mITT population).

A)

Subgroup	Patients with eradication, *n*/*N* (%) Rezafungin 400/200 mg	Caspofungin 70/50 mg	Difference (95% CI) for rezafungin − caspofungin
**Overall**	**117/161 (73)**	**116/178 (65)**	**8.6 (−1.1 to 18.3)**
**Age**			
18–<65 years	67/95 (71)	74/107 (69)	2.5 (−10.1 to 15.2)
≥65 years	50/66 (76)	42/71 (59)	16.4 (1.2 to 31.5)
65–<75 years	28/36 (78)	23/38 (61)	15.2 (−5.2 to 35.6)
≥75 years	22/30 (73)	19/33 (58)	13.3 (−8.7 to 35.4)
**Race group**			
White	71/95 (75)	70/106 (66)	9.9 (−2.6 to 22.3)
Non-White	42/61 (69)	43/68 (63)	5.3 (−10.5 to 21.1)
**Sex**			
Male	76/108 (70)	68/102 (67)	4.6 (−7.8 to 17.1)
Female	41/53 (77)	48/76 (63)	12.7 (−2.8 to 28.3)
**Geographical region**			
North/South America	26/43 (60)	30/46 (65)	−1.8 (−21.3 to 17.7)
Europe/Israel	55/67 (82)	49/76 (64)	16.9 (2.6 to 31.1)
Asia-Pacific (excl. China/Taiwan)	14/21 (67)	16/27 (59)	6.5 (−20.4 to 33.4)
China/Taiwan	22/30 (73)	21/29 (72)	1.0 (−21.8 to 23.8)
**BMI**			
<25 kg/m^2 (underweight/normal)	74/89 (83)	61/93 (66)	17.1 (4.6 to 29.6)
25–30 kg/m^2 (overweight)	20/30 (67)	35/52 (67)	−1.1 (−21.0 to 18.9)
>30 kg/m^2 (obese)	17/30 (57)	13/22 (59)	1.3 (−24.1 to 26.7)
**ANC**			
<500 cells/µL	6/9 (67)	6/10 (60)	5.3 (−35.9 to 46.4)
≥500 cells/µL	110/148 (74)	107/164 (65)	9.9 (−0.1 to 20.0)
**Renal impairment**			
≥60 mL/min (normal/mild)	64/84 (76)	66/99 (67)	10.7 (−2.3 to 23.6)
<60 mL/min (moderate/severe)	44/60 (73)	41/66 (62)	10.0 (−6.0 to 25.9)
**Modified APACHE II score**			
<10	40/57 (70)	39/57 (68)	2.1 (−14.5 to 18.7)
10–19	58/78 (74)	56/88 (64)	10.4 (−3.4 to 24.2)
<20	98/135 (73)	95/145 (66)	7.6 (−3.1 to 18.3)
≥20	18/24 (75)	18/30 (60)	12.0 (−11.7 to 35.8)
**Final diagnosis**			
Candidaemia only	95/120 (79)	92/136 (68)	12.2 (1.7 to 22.8)
IC	22/41 (54)	24/42 (57)	−1.1 (−21.0 to 18.8)
**Positive culture within window**			
Yes	45/63 (71)	47/85 (55)	15.5 (0.3 to 30.6)
No	72/98 (73)	69/93 (74)	2.4 (−9.7 to 14.6)
**Patients in ICU at randomisation**	42/55 (76)	46/77 (60)	17.1 (0.5 to 32.3)

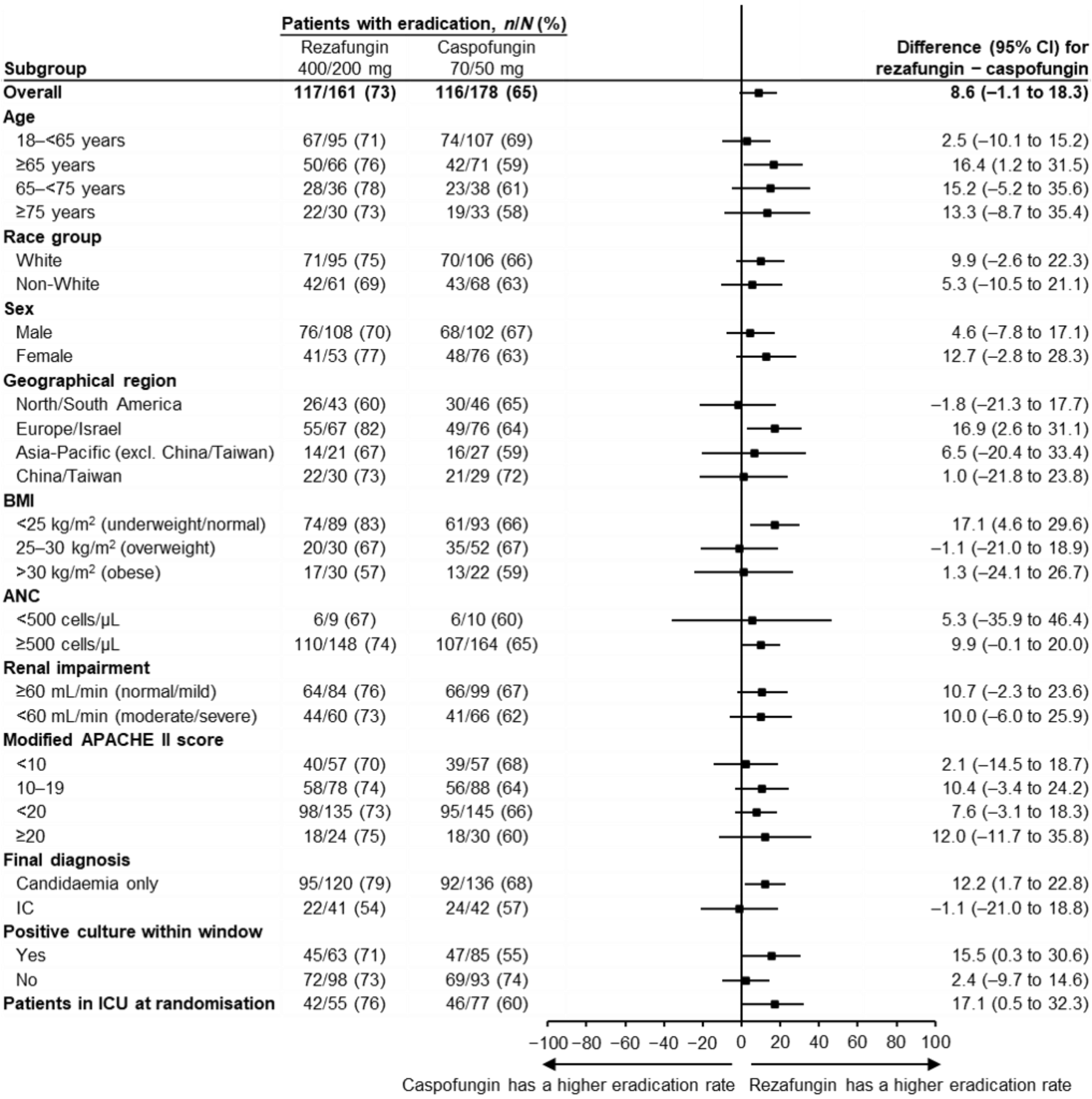

B)

Subgroup	Patients with eradication, *n*/*N* (%) Rezafungin 400/200 mg	Caspofungin 70/50 mg	Difference (95% CI) for rezafungin − caspofungin
**Overall**	**113/161 (70)**	**121/178 (68)**	**3.0 (–6.8 to 12.8)**
**Age**			
18–<65 years	62/95 (65)	79/107 (74)	–9.1 (–22.0 to 3.7)
≥65 years	51/66 (77)	42/71 (59)	18.6 (3.7 to 33.4)
65–<75 years	28/36 (78)	25/38 (66)	11.6 (–8.7 to 31.8)
≥75 years	23/30 (77)	17/33 (52)	21.6 (0.1 to 43.1)
**Race group**			
White	69/95 (73)	76/106 (72)	2.8 (–9.6 to 15.1)
Non-White	41/61 (67)	42/68 (62)	4.7 (–11.3 to 20.8)
**Sex**			
Male	73/108 (68)	72/102 (71)	0.6 (–11.5 to 12.7)
Female	40/53 (75)	49/76 (64)	9.7 (–5.8 to 25.3)
**Geographical region**			
North/South America	27/43 (63)	34/46 (74)	–9.5 (–28.7 to 9.8)
Europe/Israel	51/67 (76)	52/76 (68)	8.4 (–6.0 to 22.9)
Asia-Pacific (excl. China/Taiwan)	15/21 (71)	15/27 (56)	14.4 (–12.1 to 40.9)
China/Taiwan	20/30 (67)	20/29 (69)	–2.1 (–25.8 to 21.5)
**BMI**			
<25 kg/m^2 (underweight/normal)	66/89 (74)	63/93 (68)	5.6 (–7.6 to 18.9)
25–30 kg/m^2 (overweight)	21/30 (70)	34/52 (65)	3.7 (–16.4 to 23.8)
>30 kg/m^2 (obese)	17/30 (57)	16/22 (73)	–6.9 (–31.6 to 17.8)
**ANC**			
<500 cells/µL	5/9 (56)	6/10 (60)	–3.8 (–45.5 to 37.8)
≥500 cells/µL	107/148 (72)	112/164 (68)	4.5 (–5.6 to 14.6)
**Renal impairment**			
≥60 mL/min (normal/mild)	58/84 (69)	72/99 (73)	–3.3 (–16.6 to 10.0)
<60 mL/min (moderate/severe)	43/60 (72)	39/66 (59)	13.7 (–2.2 to 29.5)
**Modified APACHE II score**			
<10	41/57 (72)	42/57 (74)	–1.9 (–18.1 to 14.3)
10–19	57/78 (73)	58/88 (66)	9.2 (–4.4 to 22.7)
<20	98/135 (73)	100/145 (69)	3.8 (–6.8 to 14.5)
≥20	14/24 (58)	18/30 (60)	–2.3 (–26.5 to 22.0)
**Final diagnosis**			
Candidaemia only	86/120 (72)	94/136 (69)	2.0 (–9.2 to 13.2)
IC	27/41 (66)	27/42 (64)	6.2 (–12.8 to 25.2)
**Positive culture within window**			
Yes	44/63 (70)	51/85 (60)	10.4 (–4.7 to 25.5)
No	69/98 (70)	70/93 (75)	–4.3 (–16.9 to 8.4)
**Patients in ICU at randomisation**	38/55 (69)	49/77 (64)	6.4 (–10.4 to 22.0)

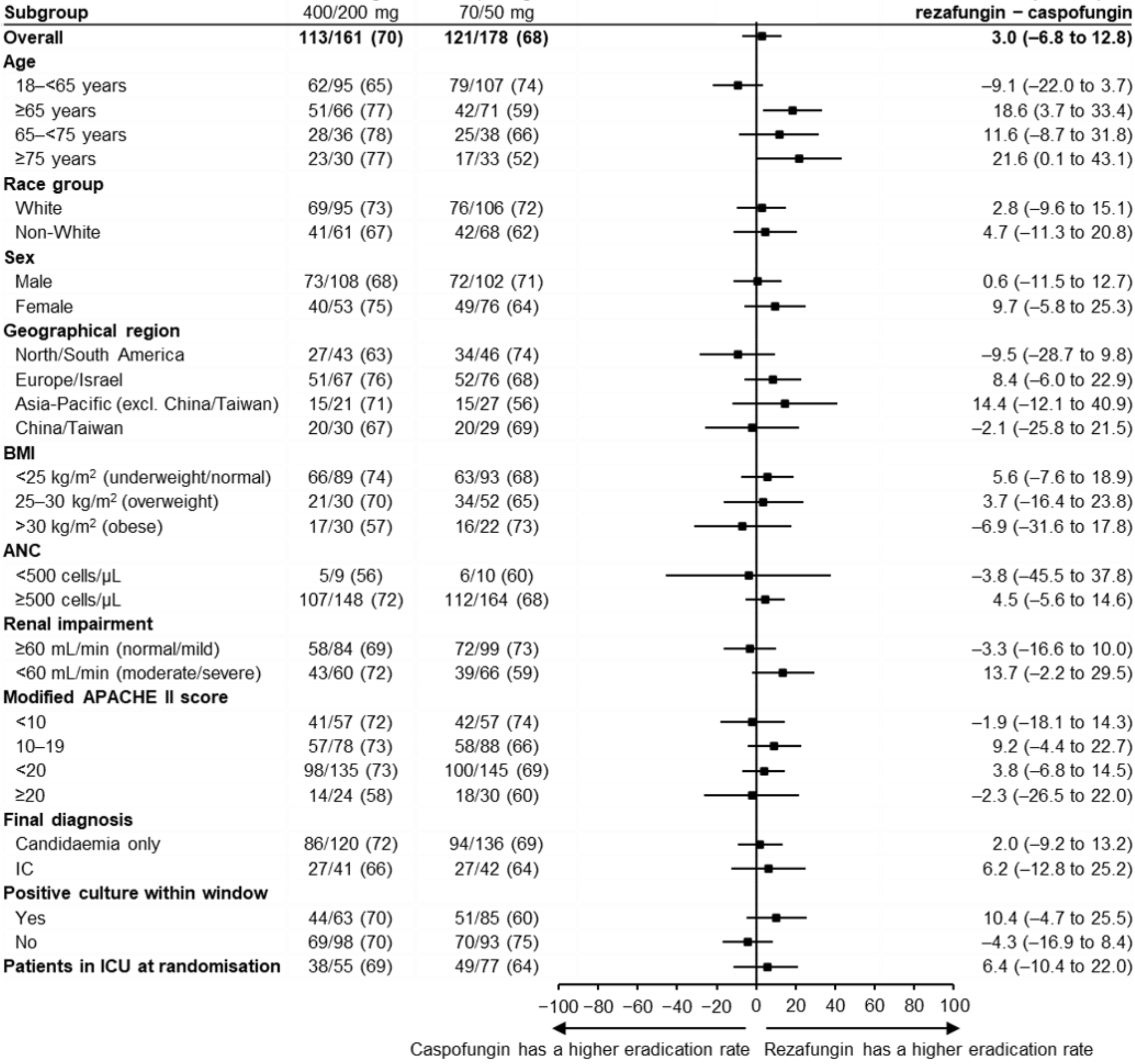

Two-sided 95% CI for the weighted difference in mycological eradication rates (rezafungin – caspofungin group) is calculated using the stratified (by study and part) Miettinen–Nurminen methodology within each subgroup. Abbreviations: APACHE, Acute Physiology and Chronic Health Evaluation; ANC, absolute neutrophil count; BMI, body mass index; CI, confidence interval; IC, invasive candidiasis; mITT, modified intent-to-treat.

References

1. G. R. Thompson, 3rd, A. Soriano, P. M. Honore, et al., "Efficacy and safety of rezafungin and caspofungin in candidaemia and invasive candidiasis: pooled data from two prospective randomised controlled trials," *The Lancet Infectious Diseases* 24 (2024): 319–328. https://doi.org/10.1016/S1473-3099(23)00551-0.

2. A. Soriano, J. B. Locke, O. A. Cornely, et al., "Clinical and mycological outcomes of candidaemia and/or invasive candidiasis by *Candida* spp. and antifungal susceptibility: pooled analyses of two randomized trials of rezafungin versus caspofungin," *Clinical Microbiology and Infection* 31 (2025): 250–257. https://doi.org/10.1016/j.cmi.2024.11.029.
